# Whence They Came

**Published:** 1881-11

**Authors:** 


### WHENCE THEY CAME.

Long before Mr. Matthew Arnold lived and wrote, Dean Swift had sung the praises of the " Two noblest things—sweetness and light." It is Swift also who wrote that " Censure is the tax a man pays to the public for being eminent," and who tells us, in his " Tale of a Tub," that " Bread is the staff of life." " Out of mind as soon as out of sight" comes from the sonnets of Lord Brooke, and it was his friend and contemporary, Sir Philip Sidney, who coined the phrase, " My dear, my better-half." Humphry Gifford, a writer of the sixteenth century, has the following:

" I cannot say a crow is white,
But needs must call a spade a spade."

Bickerstaff, a playwright as seldom read as he is often quoted, is author of the prudent admonition that " Enough is as good as a feast," and of the indisputable assertion that " One cannot have one's cake and eat it, too." From Home's " Douglas" comes the famous speech, " My name is Norval," familiar to the readers of Enfield's once celebrated but now forgotten " Speaker;" and in the same play is found the consolatory assurance that " Virtue is its own reward." "The almighty dollar" came to us from Washington Irving, and it was Beaumont and Fletcher who first taught us to speak of "money" as "the sinews of war." "How goes the enemy?" is a question often asked in the "Dramatist" of Reynolds, and "Pray, sir, what is your opinion of things in general?" is one of the "catchwords" of that impecunious sponger, Jeremy Diddler. From old Chaucer we learn that "Mordre wol out," and that it is wise to "Maken virtue of necessite." It is he, too, who wrote: "Yet in our ashen cold is fire yreken," a passage which the poet Gray must, consciously or unconsciously, have had in memory when he penned the celebrated line, "Even in our ashes live the wonted fires." It is Gray also who speaks of "Youth on the prow and Pleasure at the helm;" of "Thoughts that breathe and words that burn;" who warns us that "Favorites have no friends," and that "Where ignorance is bliss, 'tis folly to be wise." It is the shy recluse Cowper who expresses his opinion that "God made the country and man made the town," and who sings the praise of "cups that cheer but not inebriate." The light-hearted Gay instructs us that "Life is a jest, and all things show it," and it is a part of his cheerful philosophy that "While there's life there's hope."

---

Did you ever hear two married women take leave of each other at the gate on a mild evening? This is how they do it:

"Good-by."

"Good-by! Come down and see us soon."

"I will. Good-by."

"Good-by! Don't forget to come soon."

"No, I won't. Don't you forget to come up."

"I won't. Be sure and bring Sarah Jane with you next time."

"I will. I'd have brought her up this time, but she wasn't very well. She wanted to come awfully."

"Did she, now? That was too bad. Be sure and bring her next time."

"I will; and you be sure and bring the baby."

"I will I forgot to tell you he's got another tooth."

"You don't say so! How many has he now?"

"Five. It makes him awfully cross."

"I dare say it does, this hot weather. Well, good-by. Don't forget to come down."

"No, I won't. Don't you forget to come up. Good-by!"

And they separate.

